# Measurement of pulse wave velocity in normal ageing: comparison of Vicorder and magnetic resonance phase contrast imaging

**DOI:** 10.1186/s12872-016-0224-4

**Published:** 2016-02-19

**Authors:** Jehill D. Parikh, Kieren G. Hollingsworth, Vijay Kunadian, Andrew Blamire, Guy A. MacGowan

**Affiliations:** Institute of Cellular Medicine, Newcastle University, Newcastle upon Tyne, UK; Institute of Genetic Medicine, Newcastle University, Newcastle upon Tyne, UK; Cardiothoracic Centre, Freeman Hospital, Newcastle upon Tyne, UK

**Keywords:** Pulse wave velocity, Vicorder, Phase contrast magnetic resonance imaging, Ageing

## Abstract

**Background:**

Pulse wave velocity is an important measure of cardiovascular risk, and can be measured by several different techniques. We compared age-related changes in pulse wave velocity derived from carotid and femoral artery waveforms using the Vicorder device and descending thoracic aorta time velocity curves using phase contrast magnetic resonance imaging (MRI) in a group of normal healthy volunteers, without cardiovascular disease, aged between 20 and 79 years.

**Methods:**

Eighty subjects underwent same-day measurements of Vicorder and MRI pulse wave velocity measurements.

**Results:**

Both Vicorder and MRI-based pulse wave velocity measurements were significantly increased with age (*R* = 0.59 and 0.57 respectively, both *P* < 0.0001). Vicorder and MRI pulse wave velocities were also significantly related to each other (*R* = 0.27, *P* < 0.05), and Bland Altman plots showed that on average Vicorder measurements were 1.6 m/s greater than MRI. In 5 % of cases, agreement between the values of the two techniques were above and below 2 standard deviations, and these were at higher levels of pulse wave velocities. Multiple linear stepwise regression analysis confirmed highly significant relationships of both techniques to age (both *P* < 0.0001), and MRI was also significantly related to heart rate (*P* = 0.006) but Vicorder was not.

**Conclusions:**

Both Vicorder and MRI perform similarly in detecting age-related changes in pulse wave velocity. Thus, the choice of using one or the other will depend on other aspects of the investigation, such as the need for portability favouring Vicorder, or need for additional cardiovascular imaging favouring MRI.

**Trial registration:**

ClinicalTrials.Gov identifier NCT01504828

## Background

Arterial stiffness is an important determinant of cardiovascular risk, and measurement of pulse wave velocity is the most accepted measure of arterial stiffness [1–3]. This can be non-invasively measured by Doppler [4] or high fidelity pressure measuring devices to measure pulse wave velocity [5]. A quite different approach is to use phase contrast magnetic resonance imaging (MRI), measuring velocity in two or more slices of the thoracic aorta from flow waveforms [6, 7]. These inherently different techniques have distinct advantages and disadvantages. Measurements from pressure waveform techniques are relatively easily done, do not require extensive training, are quick, and can be brought to the patient. A disadvantage of the pressure techniques is that the measurement of carotid to femoral length is an external anatomical surface measure along the course of the major blood vessels and so is prone to error [8]. MRI requires significant expertise, highly trained staff, and can only be done at specialised centres. Nevertheless, the measurement of length along the aorta between the two slices where flow is measured is very accurate. Furthermore, MR imaging can be used to measure several aspects of cardiac and vascular function – so if these are already being acquired using MRI, the addition of MRI to measure vascular stiffness is relatively straightforward with advanced 4D phase contrast MRI [9].

The Vicorder device (Skidmore Medical, UK) is an inflatable cuff-based device that simultaneously measures the upstroke of carotid and femoral pulsations to calculate pulse wave velocity. It has shown good reproducibility [10] even when used by subjects with limited experience in using the device [11], and compares well with invasive measures of central blood pressure [12], and with measures of pulse wave velocity from the SphygmoCor device [10]. The purpose of this study was to compare measurements of pulse wave velocity using MRI and the Vicorder device in a population of normal subjects, without cardiovascular disease, but a wide range of ages.

## Methods

### Subjects

This study was performed as part of a larger project of ageing effects on cardiovascular function in normal subjects without a cardiovascular diagnosis, hypertension diabetes, or renal disease requiring dialysis (Cardiac Energetics and Function in Normal Human Ageing, ClinicalTrials.Gov identifier NCT01504828). Exclusion criteria were as above and also known claustrophobia. To get a consistent spread of ages we recruited subjects stratified by age ranges from 20 to 79 years. These and other general data are shown in Table 1. Informed consent was obtained for all patients, and this study was approved by a UK National Health Service Research Ethics Committee (NRES Committee North East - Newcastle & North Tyneside 1, reference number 12/NE/0057). All subjects had measurements of pulse wave velocity by the Vicorder device and Phase Contrast MRI on the same day within 2 h.

**Table 1:** Patient characteristics by age groups

	Age groups (years):					
	20–29	30–39	40–49	50–59	60–69	70–79
N	15	14	14	14	12	11
Gender (male)	5	7	6	6	8	6
Height	172 ± 8.1	174 ± 9.1	176 ± 12.5	174 ± 8.5	171 ± 9.1	164 ± 11.4
Weight	77 ± 16.6	75 ± 12.8	78 ± 9.9	77 ± 17.1	74 ± 18.1	70 ± 16.5
Glucose	4.8 ± 0.4	4.8 ± 0.5	4.9 ± 0.3	5.0 ± 0.5	5.3 ± 0.6	4.9 ± 0.5
Cholesterol	4.6 ± 0.9	4.2 ± 0.6	4.8 ± 0.9	4.9 ± 0.7	4.8 ± 0.7	5.1 ± 1.0
Triglycerides	0.9 ± 0.5	0.8 ± 0.3	0.9 ± 0.4	0.9 ± 0.7	0.9 ± 0.4	1.0 ± 0.3
HDL	1.5 ± 0.4	1.7 ± 0.3	1.5 ± 0.5	1.8 ± 0.6	1.7 ± 0.5	1.6 ± 0.3
LDL	2.7 ± 0.8	2.3 ± 0.6	2.9 ± 0.7	2.7 ± 0.8	2.7 ± 0.6	3.0 ± 0.8

Height (cm), weight (kg), all lipids and glucose measured in mmol/L

*HDL* high density lipoprotein, *LDL* low density lipoprotein

### Vicorder pulse wave velocity and other haemodynamic measurements

The Vicorder measurements were performed by trained research nurses. Patients (non fasting) were laid on an a couch in a quiet clinic room, with the head raised to approximately 30^0^, so that the skin and muscles over the carotid were relaxed though not too tense. Pulse wave velocity was measured by a cuff placed over the right carotid and the right thigh. The length between the carotid and femoral arteries was done by measuring the length between the suprasternal notch and the mid-point of the thigh cuff (Table 2). Measurements were taken until pressure waveforms over the carotid and thigh area were clear and reproducible. At a later date the lead investigator reviewed all tracings and if necessary selected only those data with clear pressure waveform upstrokes. Additional measurements with the Vicorder device were performed with a cuff placed on the right upper arm. These included oscillatory blood pressure measurement and using a global transfer function, central aortic pressures [13] and augmentation and augmentation index. Inter-observer measurements of pulse wave velocity with these methods (*N* = 32) were 7.8 ± 1.6 and 7.6 ± 1.6 m/s with mean difference of 0.2 ± 0.8 m/s (linear regression r = 0.89), and intra-observer measurements (*N* = 3) of 6.1 ± 1.0 and 6.1 ± 0.9 m/s with mean difference of 0.03 ± 0.25 m/s.

**Table 2:** Pulse wave velocities and the arterial length measured by age group

	Age groups (years):					
	20–29	30–39	40–49	50–59	60–69	70–79
Vicorder PWV	6.7 ± 0.9	6.9 ± 1.0	7.5 ± 1.2	8.0 ± 1.7	8.1 ± 1.2	9.5 ± 1.4
MRI PWV	4.5 ± 1.5	5.1 ± 0.8	6.5 ± 1.7	6.2 ± 1.6	6.8 ± 2.1	7.9 ± 1.5
Vicorder Length	63 ± 6	65 ± 6	64 ± 6	68 ± 7	67 ± 7	65 ± 9
MRI Length	10 ± 1	10 ± 2	10 ± 1	9.9 ± 8	10 ± 1	10 ± 2

*PWV* pulse wave velocity, m/sec, and lengths: cm

### Phase contrast MRI

**Fig. 1:**
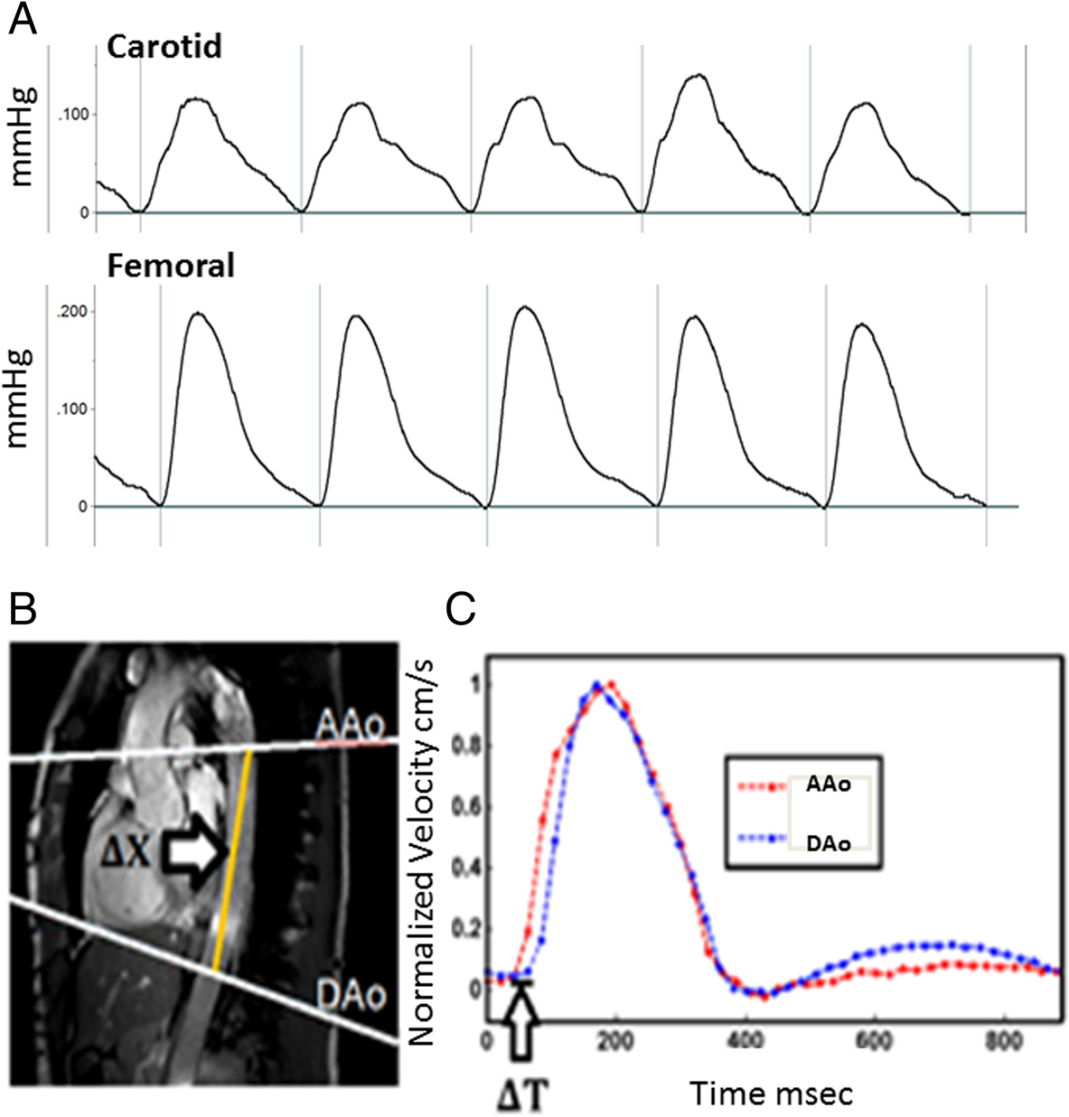
Illustration of Vicorder and phase contrast MRI techniques. In panel **a**, examples of simultaneous Vicorder carotid and femoral arterial waveforms are shown. Panel **b** shows the two slices of thoracic aorta (AAo – aortic arch, and DAo descending aorta) between which distance is measured (ΔX), and in panel **c** phase contrast MRI flow images showing ΔT which is the time delay between of the initial upstroke in flow between the two slices

Phase contrast MRI acquisitions were specifically designed to acquire time-velocity curves at two slices locations (aortic arch and descending aorta approximately 10 cm apart) to estimate transit time (ΔT) as shown in Fig. 1. Subjects were scanned using a Philips Achieva 3 Tesla scanner and a six channel cardiac coil was employed for signal reception. Phase contrast MRI data was acquired by experienced radiographers using a high temporal resolution sequence (repetition time / echo time / flip angle / number of excitations / slice thickness = 5 ms/2.9 ms/10^0^/1/8 mm, SENSE factor 2, field of view 300 mm × 225 mm, reconstructed voxel size 1.17 mm^2^, velocity encoding = 150 m/s, 44 phases, breath hold duration ~19 s) at both slice locations which were the aortic arch (AAo) and the descending (DAo) aorta (Fig. 1). Additional scout images were acquired to facilitate positioning of the phase contrast MR acquisitions and to ensure that the slices were positioned perpendicular to aorta at both locations. Q-flow analysis package (Philips, Viewform) was employed to extract time-velocity curves as shown in Fig. 1 and to estimate precise distance (ΔX) between the two slice locations. The time-velocity curves were then used to determine transit time (ΔT) and compute MR pulse wave velocity = ΔX/ΔT using an in house Matlab based program. A reproducibility analysis of this technique was performed in seven normal volunteers, repeating scans at two sessions, and within each session performing the scanning twice. For session one the values were 4.9 ± 1.6 and 4.5 ± 1.6 m/s (mean difference 0.4 ± 0.9) and session two 5.5 ± 1.6 and 5.2 ± 1.2 m/s (mean difference 0.3 ± 0.7).

### Data and statistical analysis

Data are expressed as mean ± standard deviation. Univariate associations of both Vicorder and MRI were tested with Pearson’s correlation, and multiple linear stepwise regression models to test the role of multiple parameters incorporating all the parameters from the Univariate analysis. Stepwise criteria were: Probability-of-F-to-enter < = 0.050, Probability-of-F-to-remove > = 0.100). SPSS version 21 was used for all statistics. *P* < 0.05 was considered significant.

## Results

Ninety nine patients were recruited to the main research study (between 16 and 18 subjects for each age decile from 20 to 79 years). Nine patients were excluded from this study because the Vicorder data was not adequate, usually because the carotid waveform was unclear. Six of these patients were over 50 years. Ten patients were excluded due to issues with the MR imaging. Three patients could not tolerate the MR scanning due to claustrophobia so were withdrawn. Six patients had very short transit times measured in the thoracic aorta (> 2 standard deviations from the mean). Review of these individual cases revealed that there were difficulties in positioning slices perpendicular to the aorta, particularly in those with more curved aortic arches. Five out of seven of these subjects were above 60 years of age. The remaining 80 subjects are the subject of this analysis.

### Comparison of Vicorder and MRI measures of pulse wave velocity

**Fig. 2:**
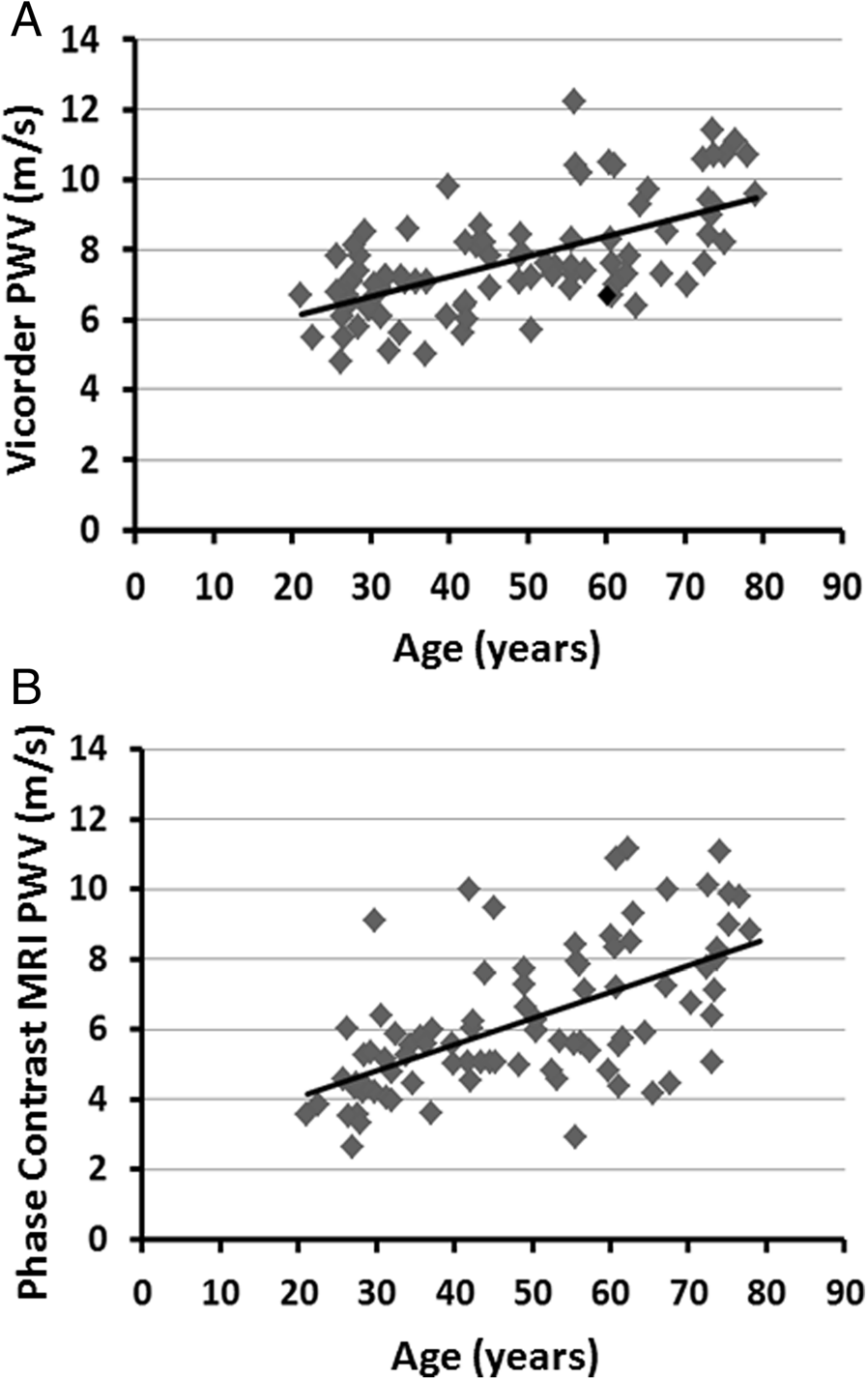
Relationship of Vicorder to age (**a**) (y = 0.055x + 5.033, R^2^ = 0.353, *R* = 0.594, *P* < 0.0001) and phase contrast MRI to age (**b**) (y = 0.067x + 2.859, R^2^ = 0.327, *R* = 0.572, *P* < 0.0001). PWV: pulse wave velocity

**Fig. 3:**
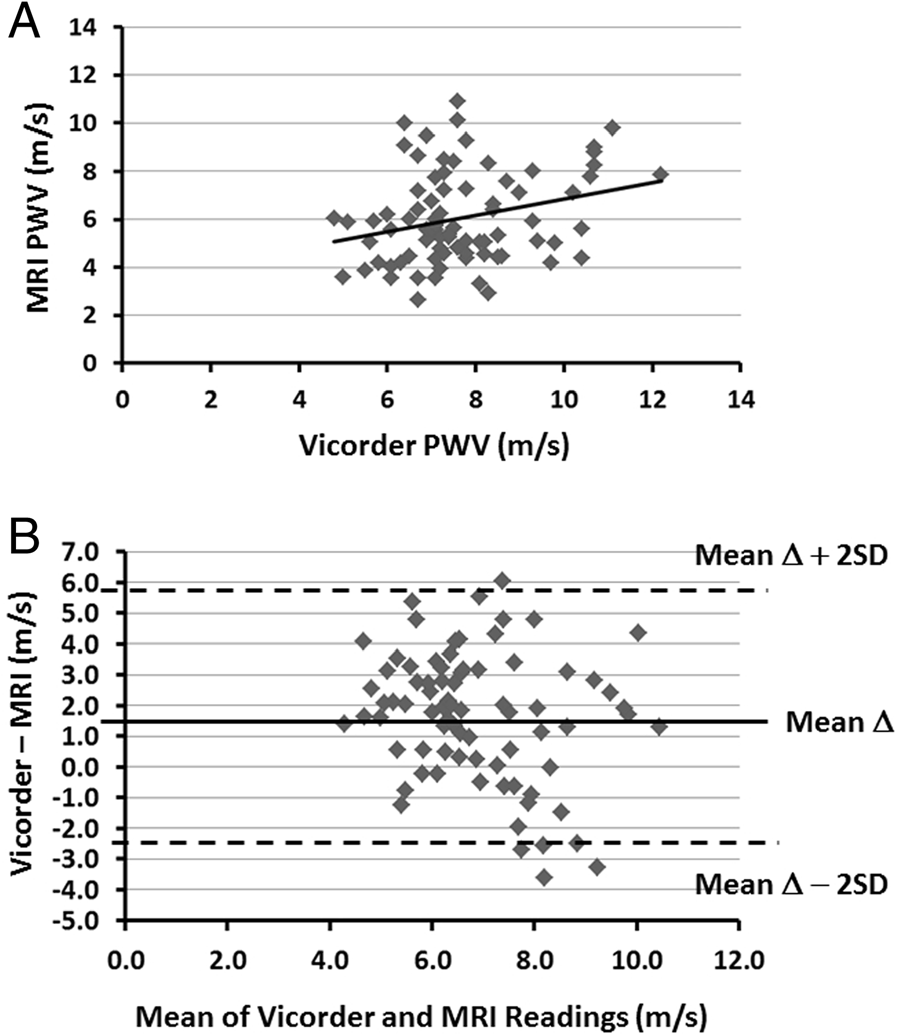
Relationship of Vicorder to phase contrast MRI (**a**) (y = 0.345x + 3.45, R^2^ = 0.073, *R* = 0.271 *P* < 0.05), and Bland Altman plot of the two techniques (**b**). On average Vicorder values are 1.6 m/s greater than MRI values. PWV: pulse wave velocity, and SD: standard deviation

Both techniques were significantly and similarly related to age (Table 2 and Fig. 2, Vicorder *R* = 0.594, and MRI *R* = 0.572), with R^2^ values in both accounting for between 35 and 33 % respectively of the variances in the model (both *P* < 0.0001). Vicorder and MRI were also significantly correlated (Fig. 3a, *R* = 0.271, 7 % of variance, *P* < 0.05). Bland Altman plot (Fig. 3b) showed that in general the mean difference between the 2 measures was 1.6 m/s greater for Vicorder. Also, particularly at higher measurements of pulse wave velocity, there were four individual cases that were greater than two standard deviations greater or less than the mean difference.

### Correlates of both measurements of pulse wave velocity

Hemodynamic data are presented by age group in Table 3. Vicorder pulse wave velocity was significantly related to all these hemodynamic variables. In general, the extent of significant correlations with these measurements were less with the MRI (Table 4), though there were highly significant correlations with aortic pulse pressure, augmentation and augmentation index. Multivariate analysis was performed separately for both techniques to compare how they related to other physiological variables (Tables 5 and 6). For Vicorder, age alone was significantly related to pulse wave velocity. For MRI age and heart rate were significantly related. In both cases the slope (Beta) against age was similar (Vicorder 0.603 and MRI 0.632).

**Table 3:** Vicorder haemodynamic data

	Age groups (years):					
	20–29	30–39	40–49	50–59	60–69	70–79
Heart Rate	64 ± 14	63 ± 14	61 ± 7	64 ± 9	59 ± 9	69 ± 14
Systolic Pressure	121 ± 11	128 ± 11	123 ± 8	124 ± 8	123 ± 12	136 ± 11
Diastolic Pressure	65 ± 7	73 ± 7	67 ± 6	68 ± 10	67 ± 9	73 ± 6
Mean Pressure	88 ± 9	95 ± 9	90 ± 7	93 ± 8	91 ± 10	100 ± 8
Pulse Pressure	60 ± 6	55 ± 5	56 ± 5	56 ± 8	55 ± 6	64 ± 9
Ao Systolic Pressure	115 ± 10	121 ± 9	120 ± 7	122 ± 8	120 ± 12	134 ± 12
Ao Pulse Pressure	50 ± 5	49 ± 5	53 ± 4	53 ± 8	53 ± 6	61 ± 9
Augmentation	6.9 ± 3.5	7.0 ± 4.3	11.2 ± 3.6	11.9 ± 3.9	13.4 ± 3.8	16.5 ± 4.4
Augmentation Index	13.7 ± 6.9	13.9 ± 8.0	21.4 ± 6.3	21.9 ± 5.0	25.5 ± 6.6	27.0 ± 6.4

Heart rate (beats per minute), all other pressures measured in mmHg, and augmentation index is a percentage

*Ao* aortic

**Table 4:** Univariate correlations

	Vicorder PWV	P =	MRI PWV	P =
Height	−0.042	0.363	−0.054	0.325
Weight	0.053	0.328	−0.044	0.354
Heart Rate	0.253	0.015	−0.213	0.035
Systolic Pressure	0.392	0.000	0.162	0.085
Diastolic Pressure	0.284	0.007	0.026	0.414
Mean Arterial Pressure	0.371	0.001	0.098	0.204
Pulse Pressure	0.286	0.007	0.224	0.028
Aortic Systolic Pressure	0.466	0.000	0.251	0.016
Aortic Pulse Pressure	0.389	0.000	0.347	0.001
Augmentation	0.358	0.001	0.419	0.000
Augmentation Index	0.290	0.006	0.377	0.000

**Table 5:** Multivariate Linear Regression Analysis

Vicorder PWV as dependent variable:	Beta	SE	P =
Age	0.603	0.009	0.000

Model R^2^ = 0.363, F = 40.5, ANOVA *P* < 0.0001

*SE* standard error

**Table 6:** Multivariate Linear Regression Analysis

MRI PWV as dependent variable	Beta	SE	P =
Age	0.632	0.010	0.000
Heart Rate	−0.256	0.014	0.006

Model R^2^ = 0.442, F = 27.7, ANOVA *P* < 0.0001

*SE* standard error

## Discussion

This study shows that when comparing two very different techniques for measuring pulse wave velocity that a) there is a very similar and highly significant relationship to age, b) there is a similar failure rate of approximately 10 % to obtain usable data in both techniques, c) that these failures and higher variances from the mean differences are greater in older subjects and those with higher values of pulse wave velocity.

### Two different techniques

The Vicorder and MRI are fundamentally two different techniques. The Vicorder measurements rely on pressure waveforms whereas MRI is based on flow. The other major difference is the region from which the pulse wave velocity is measured. The MRI is measured in a relatively small segment (10 cm) in the descending aorta, whereas the Vicorder measurements go from the carotid to femoral artery. This can explain why the Vicorder measurements are on average 1.6 m/s greater than the MRI as the peripheral arteries are stiffer than the aorta [14, 15]. The measurement of length with MRI is accurate, though with the Vicorder an anatomical external surface measure along the length of the major blood vessels is used, which is a potential source of error. Despite these differences it is reassuring that both are closely and similarly related to age.

### Comparisons with other studies

Vicorder pulse wave velocity has been assessed comprehensively in healthy subjects by Müller and colleagues [16]. In comparison with the present study, they obtained mean values of pulse wave velocity for 20–29 year olds of 5.8 ± 0.7 m/s and for 40–49 years of 7.1 ± 0.9 m/s which is slightly lower than our data particularly at the younger age (6.7 ± 0.9 and 7.5 ± 1.2 m/s respectively, Table 2). A likely explanation for this difference is in the measurement of the carotid-femoral length. Müller et al. have used the length from the suprasternal notch to the top of the femoral cuff, whereas we have taken the measurement to the middle of the cuff. Thus, our longer length will record higher pulse wave velocities. We have done this as the centre of the cuff has been shown to be where the pressure measurement is actually recorded [10]. Rogers et al [7] have looked at phase contrast MRI to measure pulse wave velocity in different regions of the aorta. Their values obtained from the proximal descending aorta which is a similar area as used in our study are comparable to our study, though values particularly in the older age groups are lower. However, this study was in a smaller cohort of patients and significant variability is seen in the older subjects. For instance, in the 20–29 year old Rogers et al. obtained a pulse wave velocity of approximately 5 m/s (compared to 4.5 ± 1.5 m/s in the present study), and for 60–69 years old approximately 9 m/s compared to 6.8 ± 2.1 m/s in the present study. In a larger study of 162 normal subjects Hickson and colleagues [17] have compared the SphygmaCor and Vicorder devices and phase contrast MRI. Their values of phase contrast MRI pulse wave velocity in the descending thoracic aorta are very similar to the current study. They did however show a closer relationship of Vicorder pulse wave velocity measurements to phase contrast MRI measurements than in the current study (*r* = 0.64 vs *r* = 0.27 in current study). This higher level of correlation may be due to the larger number of subjects studied and the measurement of pulse wave velocity along the whole length of the descending aorta by phase contrast MRI. Nevertheless Bland Altman plots show a similar pattern to the current study with higher overall mean values for Vicorder, and individual points outside two standard deviations particularly at higher levels of pulse wave velocity. Thus, the values of both Vicorder and MRI pulse wave velocity obtained in this study match very closely to published data.

### Clinical significance

There are two issues of particular clinical significance with these data. Firstly, measurement of pulse wave velocity is recommended in the 2013 European Society of Cardiology/European Society of Hypertension guidelines for assessment of cardiovascular risk [18], though there are continuing issues regarding clinical application that have been recently highlighted [19]. The issue that is most relevant to this study is that there are a myriad of devices that can measure pulse wave velocity and that these are not necessarily interchangeable. In that regard, our data is very important comparing these two techniques. In particular, as MR imaging becomes more widely adapted in clinical cardiovascular diagnostics, how this performs compared to other techniques is very important. The second issue of clinical significance is that with both techniques there were approximately 10 % of cases in which data was not obtainable or usable. This was more prevalent in older subjects. One of the advantages with the Vicorder system is that it is easy to use, and so in this study all examinations were performed by research nursing staff. However, despite this there are cases where the carotid waveform is simply not clear enough to identify the upstroke and therefore not usable, and this appears higher than previously reported [11]. For the MRI scanning there were similarly issues with both claustrophobia and tolerating the scan, and also poor data quality which is predominantly in the older subjects. The scans were performed by experienced radiographers, though there were issues with obtaining scans perpendicular to the descending aorta in older subjects as a result of increased aortic curvature. As these techniques move from a purely research domain to clinical tools, these data are important to understand how these techniques perform. Our data suggests that assessing pulse wave velocity in older subjects requires careful attention to these technical issues to avoid loss of potentially important data, and more information is required to determine how other techniques perform in these older subjects.

### Limitations

These data are in normal subjects, so in subjects with cardiovascular disease with increased vascular stiffness the relationships described may be altered, and also there may be additional issues with acquiring high quality data. Gender differences have been described with measures of vascular stiffness [20], but this study was not statistically powered to detect gender differences. There are other methods to measure pulse wave velocity with MRI. For instance, other areas of the aorta can be assessed using phase contrast MRI. Hickson and colleagues [17] have shown that the abdominal aorta has slightly higher rates of age-related increases in pulse wave velocity relative to the descending thoracic aorta. Other phase contrast techniques include determination of flow-area [21], and cross-correlation methods [22] (where flow is determined at several points along the descending aorta). Ibrahim et al. [23] have shown that the transit time (as used in this study) and cross-correlation methods resulted in more reproducible measurements compared to the flow-area method. Our MRI reproducibility studies have more variation than the data published by Ibrahim et al. explained by their much larger study number. A dependency of heart rate on augmentation index has been reported for the SphygmoCor device [24]. As there is no published data on this issue with the Vicorder device, we have not normalised data to heart rate.

## Conclusions

Both the Vicorder device and phase contrast MRI measurements of pulse wave velocity are similarly and strongly related to age. The decision on whether to use one or the other in studies of vascular ageing will thus be related to other factors – such as the portability and ease of use of the Vicorder device, or need for additional cardiovascular MR imaging.

## Abbreviations

MRI: magnetic resonance imaging
AAo: arch of aorta
DAo: descending aorta
HDL: high density lipoprotein
LDL: low density lipoprotein
PWV: pulse wave velocity
Ao: aortic
SE: standard error
